# The scientific progress and prospects of artificial intelligence in digestive endoscopy: A comprehensive bibliometric analysis

**DOI:** 10.1097/MD.0000000000031931

**Published:** 2022-11-25

**Authors:** Pei-Ling Gan, Shu Huang, Xiao Pan, Hui-Fang Xia, Mu-Han Lü, Xian Zhou, Xiao-Wei Tang

**Affiliations:** a Department of Gastroenterology, the Affiliated Hospital of Southwest Medical University, Luzhou, China; b Department of Gastroenterology, the People’s Hospital of Lianshui, Huaian, China.

**Keywords:** artificial intelligence, digestive endoscope, visualization, VOSviewer. CiteSpace

## Abstract

Artificial intelligence (AI) has been used for diagnosis and outcome prediction in clinical practice. Furthermore, AI in digestive endoscopy has attracted much attention and shown promising and stimulating results. This study aimed to determine the development trends and research hotspots of AI in digestive endoscopy by visualizing articles. Publications on AI in digestive endoscopy research were retrieved from the Web of Science Core Collection on April 25, 2022. VOSviewer and CiteSpace were used to assess and plot the research outputs. This analytical research was based on original articles and reviews. A total of 524 records of AI research in digestive endoscopy, published between 2005 and 2022, were retrieved. The number of articles has increased 27-fold from 2017 to 2021. Fifty-one countries and 994 institutions contributed to all publications. Asian countries had the highest number of publications. China, the USA, and Japan were consistently the leading driving forces and mainly contributed (26%, 21%, and 14.31%, respectively). With a solid academic reputation in this area, Japan has the highest number of citations per article. Tada Tomohiro published the most articles and received the most citations.. *Gastrointestinal endoscopy* published the largest number of publications, and 4 of the top 10 cited papers were published in this journal. “The Classification,” “ulcerative colitis,” “capsule endoscopy,” “polyp detection,” and “early gastric cancer” were the leading research hotspots. Our study provides systematic elaboration for researchers to better understand the development of AI in gastrointestinal endoscopy.

## 1. Introduction

Artificial intelligence (AI) has become a term related to automation software designed to replace human cognitive processes. Contemporary AI is a complex algorithm that can complete tasks without explicit instructions. This ability is also called machine learning (ML). ML models are trained using various datasets to extract and transform features, while realizing the object of classification and prediction by self-learning. “Deep learning (DL)” is a superior form of ML, which means that multiple algorithms exist in complex layers.^[1–3]^ “Artificial Neural Network^[4]^” or “Convolutional Neural Network (CNN)^[5]^” are terminology for this type of layer. These algorithms are intertwined with each other, can identify information characteristics that a single algorithm may not be able to complete independently, and exponentially improve the learning ability of the algorithm.

AI has been widely used in various domains. Its applications include ophthalmoscopy,^[6]^ electronic colposcope,^[7]^ cardiology,^[8]^ and nephrology.^[9]^ However, there have been no previous bibliometric analyses of AI in digestive endoscopy. Herein, we analyzed its application in endoscopy with an emphasis on gastroenterology using bibliometric methods and facilitated researchers to obtain a good comprehension of research hotspots and deficiencies.

## 2. Methods

### 2.1. Search strategies

**Figure 1.**
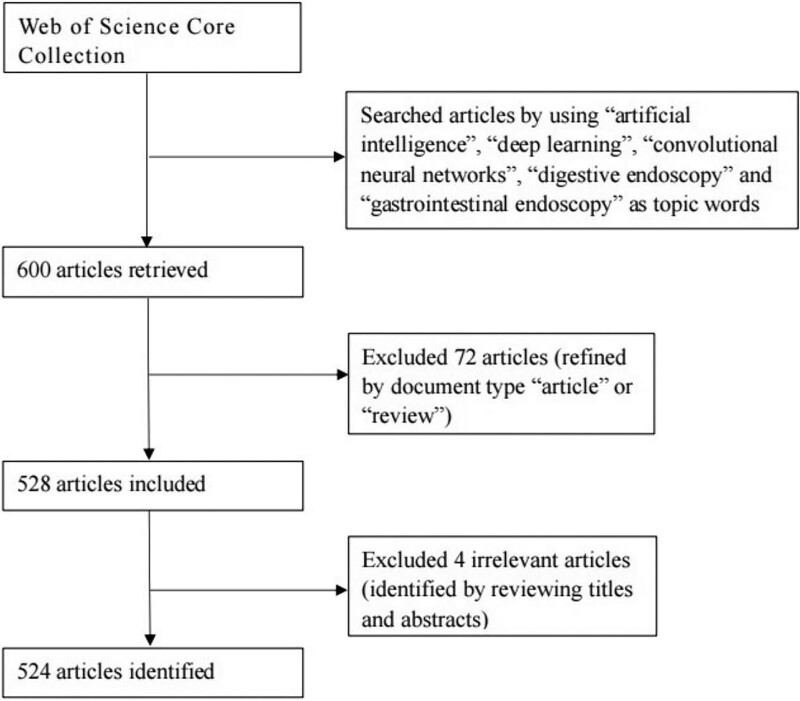
A flowchart representing retrieval strategies for artificial intelligence in digestive endoscopy articles from the Web of Science Core Collection database and the inclusion criteria for the study.

Data were retrieved from the Web of Science Core Collection (WoSCC) within 1 day on April 25, 2022, by using “artificial intelligence,” “deep learning,” “convolutional neural networks,” “digestive endoscopy,” and “gastrointestinal endoscopy” as topic words. The search strategy used were the following: “(TS = (artificial intelligence)) AND TS = (digestive endoscopy)” OR “(TS = (artificial intelligence)) AND TS = (gastrointestinal endoscopy)” OR “(TS = (deep learning)) AND TS = (gastrointestinal endoscopy)” OR “(TS = (deep learning)) AND TS = (digestive endoscopy)” OR “(TS = (convolutional neural networks)) AND TS = (digestive endoscopy)” OR “(TS = (convolutional neural networks)) AND TS = (gastrointestinal endoscopy).” There were no limitations in terms of language or publication year. The search resulted in 600 records; 72 records were eliminated, and only original articles and reviews were included. We excluded 4 irrelevant articles by reviewing titles and abstracts. We obtained 524 records for this study. A flowchart representing the retrieval strategy is shown in Figure 1.

### 2.2. Data collection and analysis

All retrieved records from WoSCC were downloaded and bibliometric indicators were analyzed, including the number of publications (continents, countries, institutions, journals, and authors) and the frequency of citations. Journal Citation Reports was used to obtain the impact factor (IF) and eigenfactor™ score (EF). The data were then converted into Microsoft Excel 2016, VOSviewer, and CiteSpace for further analysis.

EF is an alternative metric for evaluating the significance of a journal.^[10]^ While the IF measures the journal’s citation numbers by the number of publications in the preceding 2 years, the EF is calculated based on the number of publications in the past 5 years. Unlike IF, EF eliminates self-citations and assigns a weight to each citation. The scores of all journals in Journal Citation Reports sum to 100, and each journal’s score was scaled. For instance, if a journal’s score is 1.0, it has 1% of all indexed journals’ total influence. Herein, we listed both IF and EF, which identified journals’ widespread impact in this scientific field.

Publication output, IF and EF, citations per article, and total citations of each country, institution, journal, and author were analyzed and plotted using Microsoft Excel 2016, and data were collated on basic characteristics of publications and citations.

VOSviewer analyzes collaborative relationships between countries, institutions, and authors of highly co-cited references by creating a visual map.^[11]^ Furthermore, VOSviewer can divide keywords with a high co-occurrence frequency into different clusters and simultaneously color them according to the time course of the diverse clusters. A co-occurrence analysis can theoretically identify research hotspots and trends. We selected “all keywords” as the unit of analysis. Co-authorship analysis identifies the research output. We selected “authors,” “organizations, and “countries” as the units of analysis.

CiteSpace adopts a time-slicing technique to create a timeline of network models and integrates these individual networks to produce an overview network for the systematic analysis of relevant publications.^[12,13]^ In this study, CiteSpace was used to perform a co-citation analysis and construct a timeline view of the co-cited references. The “years per slice” and “top N per slice” values were set to 1 and 50, respectively. Thus, we can define the rise and period of specific cluster fields.

## 3. Results

### 3.1. Publication output and temporal trend

**Figure 2.**
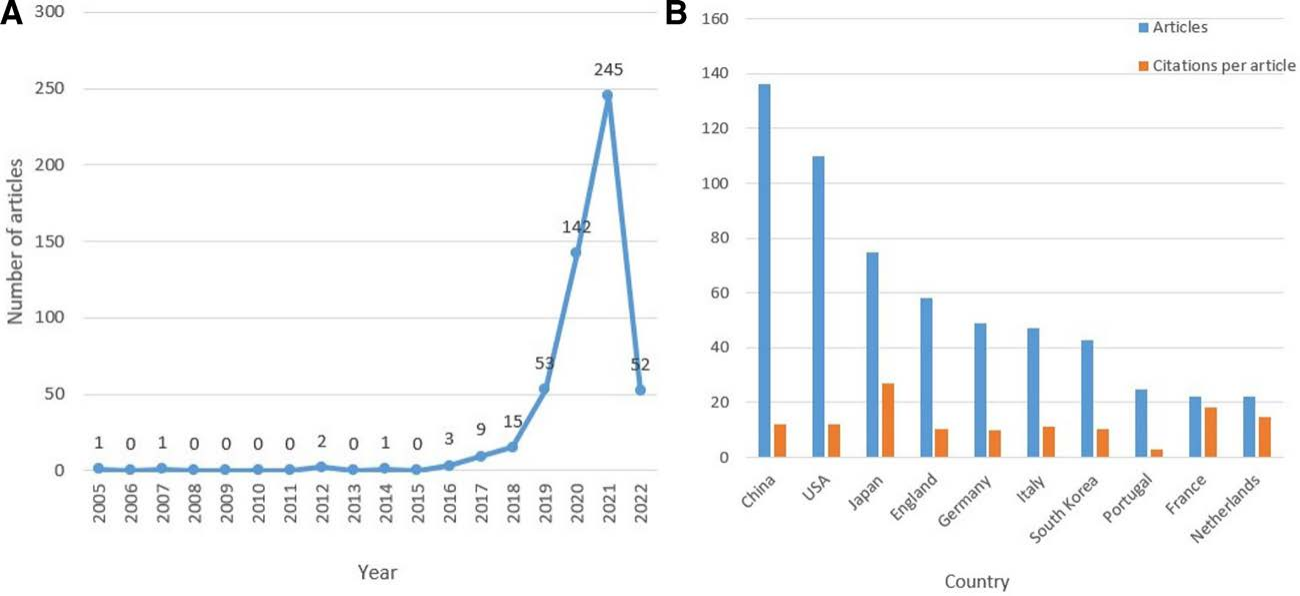
Trends in the number of publications and analysis of countries in artificial intelligence in digestive endoscopy field. (A) The annual worldwide publication output. (B) The total number of publications and citations per article for the top 10 countries.

In total, 524 papers met the inclusion criteria. The years of publication were from 2005 to 2022. Table 1 lists the top-10 cited articles in descending order based on the number of citations, and Table S1 lists the top-100 cited articles (see Table S1, Supplemental Digital Content, http://links.lww.com/MD/H986, which includes detailed information). The number of the top-10 articles ranged from 97 to 255. The number of publications in this area increased steadily until 2018, with significant growth occurring in the 4 years following 2018. To date, 52 articles have been published by 2022 (Fig. 2A). However, this figure did not reflect the number of publications throughout the year.

**Table 1:** The top-10 cited articles of WoSCC bibliometrics in artificial intelligence in digestive endoscopy field.

Rank	First author	Journal	Title	Number of citations (WoSCC)	Type of articles
1	Hirasawa, T	Gastric Cancer. 2018;21(4):653–660	Application of artificial intelligence using a convolutional neural network for detecting gastric cancer in endoscopic images	255	Article
2	Wang, P	Gut. 2019;68(10):1813–1819	Real-time automatic detection system increases colonoscopic polyp and adenoma detection rates: a prospective randomized controlled study	248	Randomized Controlled Trial
3	Mori, Y	Ann Intern Med. 2018;18;169(6):357–366	Real-Time Use of Artificial Intelligence in Identification of Diminutive Polyps During Colonoscopy A Prospective Study	195	Evaluation Study
4	Horie, Y	Gastrointest Endosc. 2019;89(1):25–32	Diagnostic outcomes of esophageal cancer by artificial intelligence using convolutional neural networks	153	Article
5	Le Berre, C	Gastroenterology. 2020;158(1):76–94.e2	Application of Artificial Intelligence to Gastroenterology and Hepatology	129	Review
6	Zhu, Y	Gastrointest Endosc. 2019;89(4):806–815.e1	Application of convolutional neural network in the diagnosis of the invasion depth of gastric cancer based on conventional endoscopy	127	Article
7	Wang, P	Gut. 2019;68(10):1813–1819	Application of Convolutional Neural Networks in the Diagnosis of Helicobacter pylori Infection Based on Endoscopic Images	124	Randomized Controlled Trial
8	Maeda, Y	Gastrointest Endosc. 2019;89(2):408–415	Real-time artificial intelligence for detection of upper gastrointestinal cancer by endoscopy: a multicentre, case-control, diagnostic study	110	Randomized Controlled Trial
9	Aoki, T	Gastrointest Endosc. 2019;89(2):357–363.e2	Automatic detection of erosions and ulcerations in wireless capsule endoscopy images based on a deep convolutional neural network	110	Article
10	Wu, L.	Gut, 68(12), 2161–2169	Randomised controlled trial of WISENSE, a real-time quality improving system for monitoring blind spots during esophagogastroduodenoscopy	97	Randomized Controlled Tria

WoSCC = Web of Science Core Collection.

### 3.2. Distribution by country, institution, and authors

**Figure 3.**
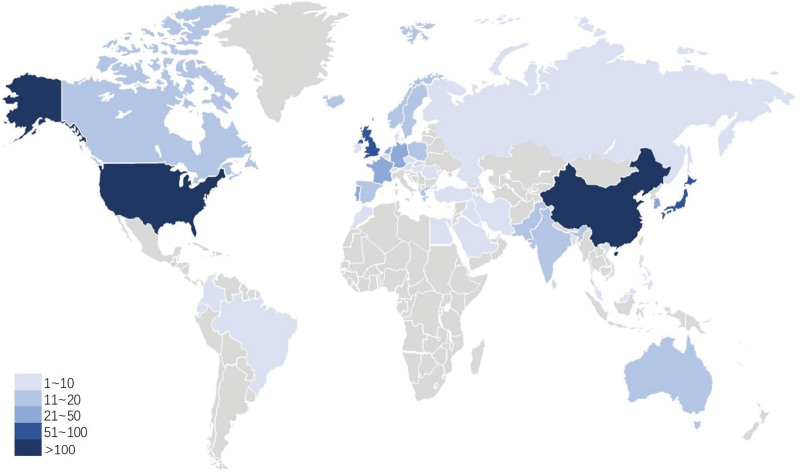
Worldwide distributions of the Web of Science Core Collection publications on artificial intelligence in digestive endoscopy field.

Fifty-one countries (see Table S2, Supplemental Digital Content, http://links.lww.com/MD/H987, which includes detailed information on all countries) and 994 institutions contributed to all publications. Figure 2B lists the number of citations per article and the total number of published articles for the top 10 countries. The worldwide distribution of publications is shown in Figure 3. Asia housed more than half the papers. China had the largest production, with 136 (26%) documents, followed by the USA (110, 21%), Japan (75, 14.31%), England (58, 11.07%), and Germany (49, 9.35%). Japan had 2040 citations, ranking first among all countries included in the analysis. Furthermore, its citation/article ratio (27.2) was far higher than that of other listed countries. Although China had published more articles and ranked first, it had a relatively low citation/article ratio.

**Figure 4.**
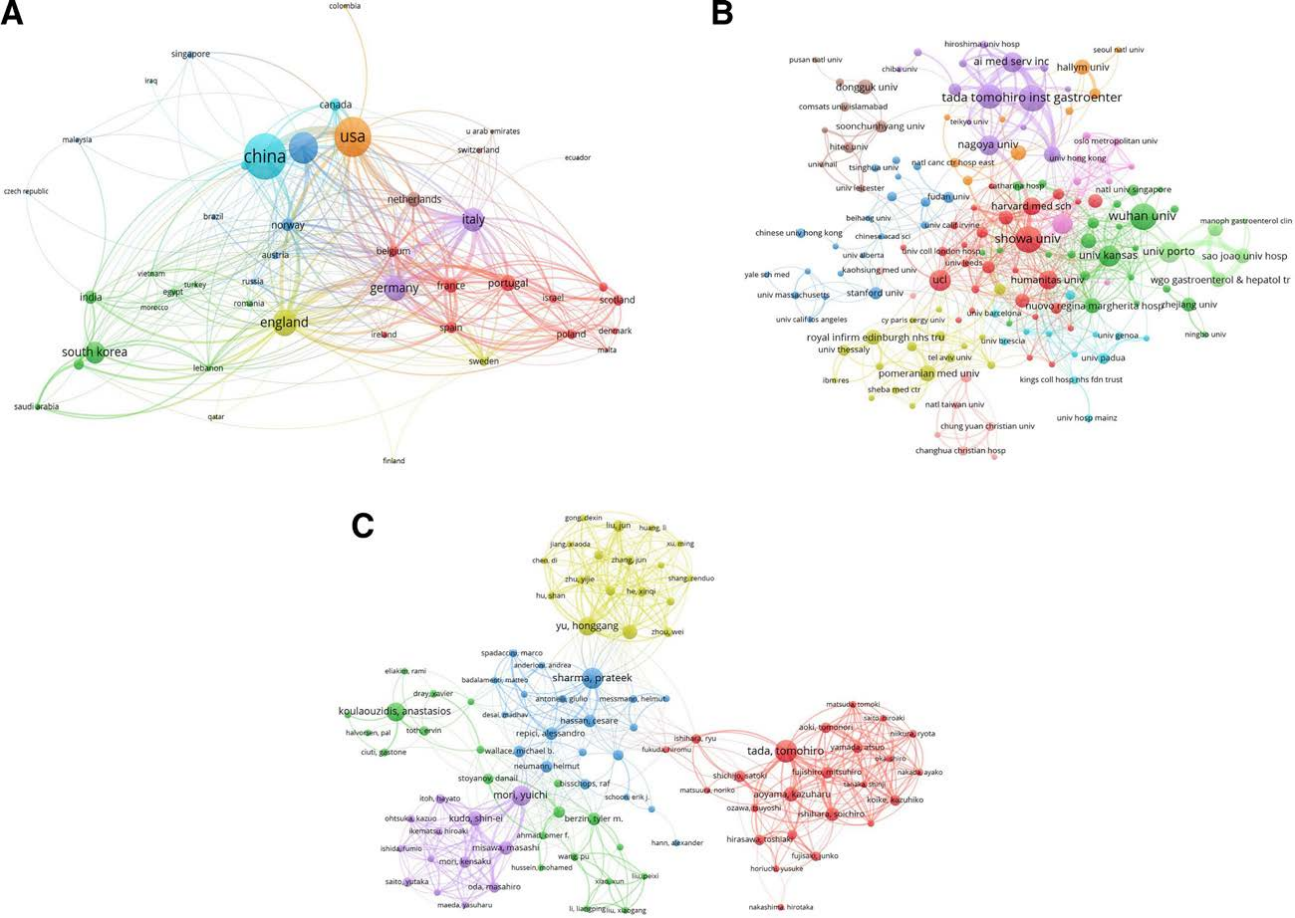
Scientific influence of artificial intelligence in digestive endoscopy worldwide. (A) Network plot of influential countries. (B) Network plot of influential institutions. (C) Network plot of influential authors.

To analyze international collaborations, we used VOSviewer to construct a visualization map of research publications related to AI in digestive endoscopy. Figure 4A shows partnerships between countries. Nodes with high co-occurrence were stained with the same color. Nodes with similar colors formed 1 cluster, indicating that they had closer partnerships. The different widths of the colored lines indicate different scales of collaboration. The USA had the highest total link strength, suggesting that it cooperated the most with other countries worldwide. China collaborated the most with the USA.

Table 2 lists the most productive institutions. Figure 4B shows the collaborations among the institutions. Purple, red, and green were the 3 clusters that contributed the most. The purple cluster was led by the Tada Tomohiro Institute of Gastroenterology, collaborating mostly with AI Medical Service Incorporated. The red cluster was represented by Showa University, followed by University College London, Harvard Medical School, and Humanitas University. The green cluster was led by Wuhan University, followed by the University of Kansas, and the University of Porto.

**Table 2:** Institutions of origin with top-cited articles.

Rank	Institutions	Number of articles	Number of Citations	Country
1	Tada Tomohiro Institute of Gastroenterology and Proctology	19	995	Japan
2	Showa University	19	663	Japan
3	Wuhan University	19	198	China
4	The University of Tokyo	17	951	Japan
5	University of Kansas	14	114	USA
6	University College London	14	107	England
7	University of Porto	14	48	Portugal
8	AI Medical Service Incorporated	13	657	Japan
9	Nagoya University	13	591	Japan
10	University of Oslo	13	163	Norway

In total, 2589 authors contributed to all articles retrieved. The most productive authors are listed in Table 3. Tada Tomohiro published 20 articles, ranking first in the number of publications, followed by Sharma Prateek (18 articles), Mori and Yuichi (17 articles), and Koulaouzidis and Anastasios (16 articles). A network visualization map of the coauthors is shown in Figure 4C.

**Table 3:** Authors with top-cited articles.

Rank	Author	Number of articles	Number of citations
1	Tada, Tomohiro	20	999
2	Sharma, Prateek	18	176
3	Mori, Yuichi	17	586
4	Koulaouzidis, Anastasios	16	191
5	Yu, Honggang	15	101
6	Aoyama, Kazuharu	11	863
7	Kudo, Shin-EI	11	475
8	Wu, Lianlian	11	76
9	Ishihara, Soichiro	10	829
10	Misawa, Masashi	10	474

### 3.3. Distribution by journal

All the papers included in the analysis were published in 188 professional academic journals. The most productive journals are listed in Table 4. *Gastrointestinal Endoscopy* published the most articles in this field (30 publications), with an IF of 9.427 and an EF of 0.03080. This was followed by *the World Journal of Gastroenterology* (27 publications) and *the Digestive Endoscopy* (25 publications). The most frequently cited journal was *Gastrointestinal Endoscopy* (945 citations). *Digestive Endoscopy* (298 citations) was the next most frequently cited journal. In terms of the disciplinary categories of journals, AI research in digestive endoscopy is not limited to gastroenterology or hepatology. Research in this field may require multidisciplinary collaborative research involving telecommunication and imaging.

**Table 4:** Journals with the top-cited articles of artificial intelligence in digestive endoscopy field.

Rank	Journal	Number of articles	Citations	IF	EF	Category by JCR
1	Gastrointestinal Endoscopy	30	945	9.427	0.03080	Gastroenterology & Hepatology
2	World Journal of Gastroenterology	27	151	5.742	0.05072	Gastroenterology & Hepatology
3	Digestive Endoscopy	25	298	7.559	0.00611	Gastroenterology & Hepatology; Surgery
4	Journal of Gastroenterology and Hepatology	18	131	4.029	0.01536	Gastroenterology & Hepatology
5	Diagnostics	14	13	3.706	0.00318	Medicine, General & Internal
6	Endoscopy International Open	13	131	0.52	0.00775	Gastroenterology & Hepatology
7	Journal of Clinical Medicine	13	116	4.242	0.03135	Medicine, General & Internal
8	Ieee Access	11	154	3.367	0.015383	Telecommunications
9	Clinical Endoscopy	11	105	0.41	0.00245	Gastroenterology & Hepatology
10	Best Practice & Research Clinical Gastroenterology	11	6	3.043	0.00364	Gastroenterology & Hepatology

EF = Eigenfactor™ score, IF = impact factor, JCR = journal citation reports, WoSCC = Web of Science Core Collection.

### 3.4. Analysis of keywords

**Figure 5.**
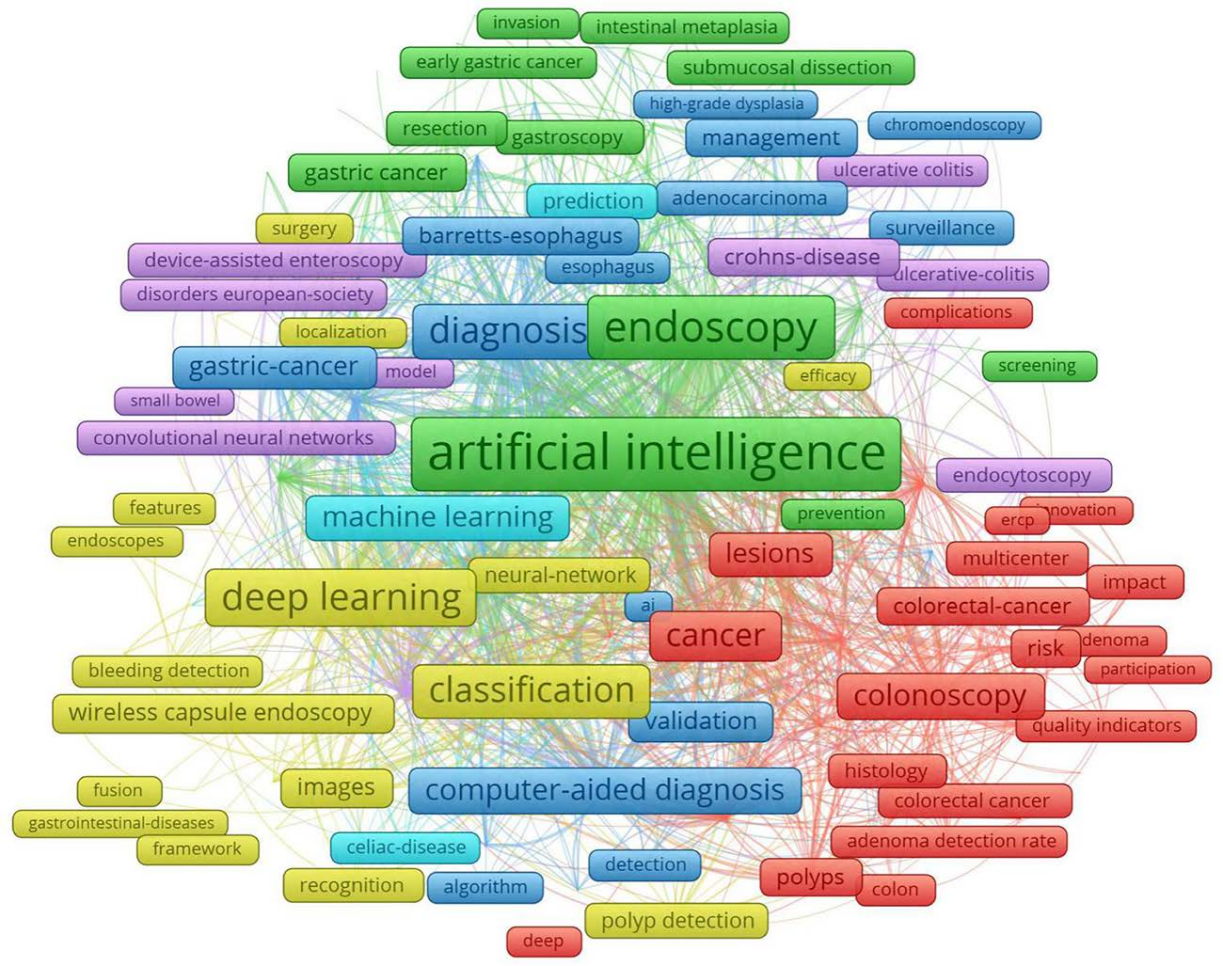
Network plot of influential keywords in artificial intelligence in digestive endoscopy research.

VOSviewer was used to analyze the keywords and identify hotspots. A network visualization of the keywords is shown in Figure 5. The size of the circles represents the keyword occurrence frequency. The larger the circle, the more frequently keywords occurred. As the graph showed, “artificial intelligence,” “endoscopy,” “deep learning,” “classification,” “cancer,” “capsule endoscopy,” and “diagnosis” were the most influential keywords.

### 3.5. Analysis of co-cited references

**Figure 6.**
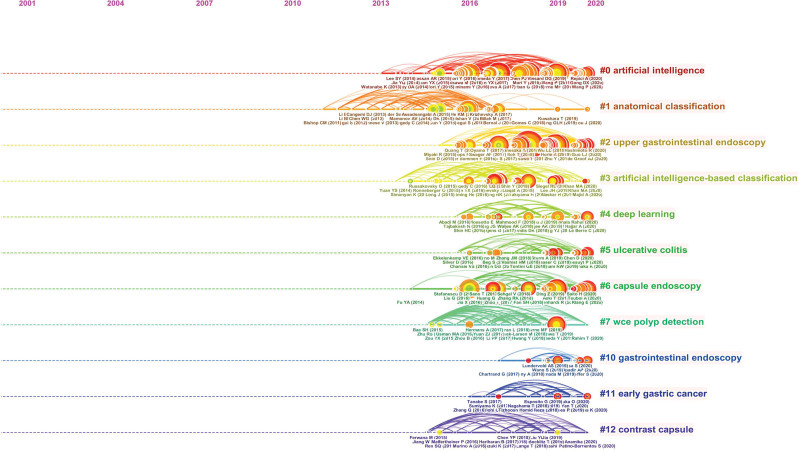
A timeline visualization of the largest clusters.

Clusters, along with horizontal timelines, are depicted in Figure 6, which is a timeline visualization in CiteSpace. Each cluster is presented from left to right. The publication time legend is displayed at the top. The clusters were arranged vertically and in a declining order of size. The largest cluster is presented at the top. The co-citation linkages are indicated by the stained curves. Large-sized nodes were especially concerning, because they were highly cited. Below each timeline are the most cited references for a given year. The clusters were numbered from 0. Cluster #0 represents the largest cluster.^[14]^ As shown in the timeline overview, the largest cluster in this study was #0 AI. The clusters were followed by #1 anatomical classification, #2 upper gastrointestinal endoscopy, #3 AI-based classification, #4 deep learning, #5 ulcerative colitis, #6 capsule endoscopy, and #7 WCE polyp detection, indicating a significant research interest and direction in recent years.

**Figure 7.**
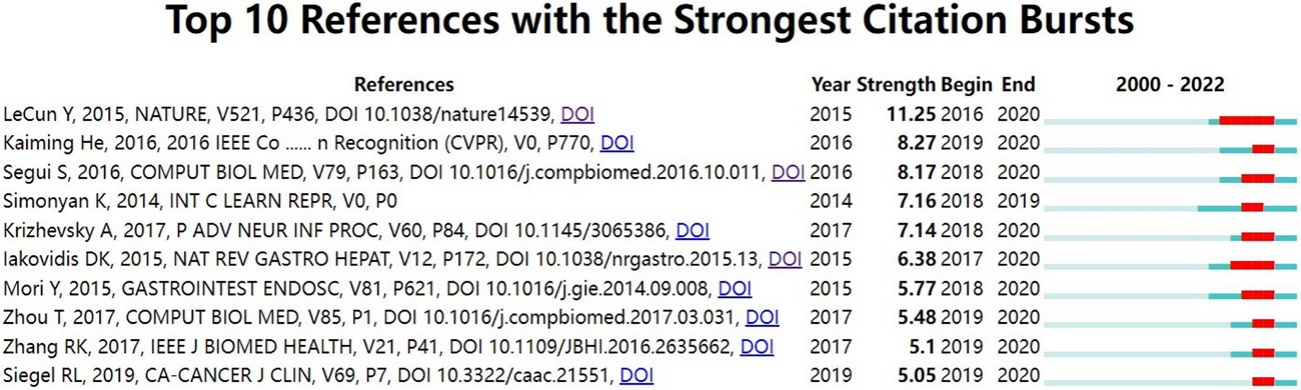
The top 10 references with the strongest citation bursts.

Figure 7 lists the top 20 references with the strongest citation burst. Burst references refer to references heavily cited by other articles over a period of time. From the bursts of cited references, the literature from 2014 to 2019 has opened the research hotspot of AI in GI endoscopy. References with citation bursts first appeared in 2016. However, most of the citation bursts ended in 2019 or 2020. This may be related to the shock of the COVID-19.

The top 10 references with the strongest citation burst strengths are presented in Figure 7. Papers with high citation bursts have received much attention from researchers. Hence, references with high citation bursts represent emerging trends or topics.^[14]^ Overall, the burst strength of the top 10 references ranged from 5.05 to 11.25, while the endurance strength ranged from 2 to 5 years, as indicated by the number of red squares. Among the top 10 papers, the citation burst of 9 publications ended in 2020; therefore, they reflect the most recent research topics. The first paper^[1]^ was published in 2015, and the citation burst lasted for 5 years (2016–2020). This paper, published in *Nature*, provides a scientific review of the current state and future prospects of deep learning. In 2016, He et al^[15]^ presented a learning framework to ease the training of networks that are substantially deeper than those used previously. This study had the second strongest citation burst and contributed to the application of information technology in the medical field. In a paper with the third-highest citation burst,^[16]^ the authors described a system for small intestine characterization of wireless capsule endoscopy based on deep convolutional neural networks. This study was published in 2016 and had a burst strength of 8.17, and its burst lasted for 3 years from 2018 to 2020.

## 4. Discussion

Up to date, the application of AI has emerged as a useful tool for the diagnosis of digestive diseases. For instance, AI can be used for intelligent quality control of bowel preparation in each segment of the colon, to identify and judge the preparation quality automatically, and to evaluate the bowel preparation status in real time.^[17]^ Studies have shown that colonoscopic withdrawal time can affect adenoma detection rate during colonoscopy screening.^[18]^ AI can be used for intelligent quality control of colonoscopic withdrawal time. Through image dataset training, the system automatically determined the start and end points of colonoscopy withdrawal. AI can also facilitate the detection of small lesions, reduce their omission of small lesions, and reduce the blind area during endoscopic examination. Doctors are prone to visual fatigue during workloads. AI can compensate for the insufficiency of human visual observation and achieve complete endoscopic operation. AI in endoscopy technology is of great significance for the development of the medical industry. This will give birth to a new generation of medical equipment in the field of digestive endoscopy.

From the retrieved data, we can conclude that AI studies on endoscopy have been booming since 2018. This phenomenon is inseparable from the historical processes of AI. In 1956, the term “artificial intelligence” was formally proposed at Dartmouth College.^[19]^ In 2006, the DL algorithm training multi-layer neural networks emerged, which brought a spring to AI.^[20]^ The DL algorithm was first applied in speech recognition, and then widely used in images and vision. In 2012, Krichevsky et al^[21]^ proposed a CNN with a 5-roll-up layer and 2-layer full connection layer implemented using a compute unified device architecture. The emergence of this algorithm has provided a starting point for the application of AI to computer vision. Theory and technology have become more mature, and their application field is also expanding. Thus, the Japanese government revised the “Japan Revitalization Strategy” adopted by the cabinet in June 2014, which proposes to promote the “robot-driven new industrial revolution.” In October 2016, the United States issued a national strategic plan for AI research and development. On July 20, 2017, China issued a development plan for a new generation of AI. From these policies, we can see that a variety of countries led by Japan are seizing the commanding heights of AI technology, which further promoted AI development in endoscopy. The publication output is expected to increase considerably by 2021.

Japan’s robotics industry accounts for far more economic growth than other countries in the world. In the past 30 years, Japan has been known as the “robot superpower,” which has the largest number of robot users, equipment, and service manufacturers globally. Furthermore, the Tada Tomohiro Institute of Gastroenterology and Proctology in Japan is the most productive institution worldwide and closely collaborates with many others. On the other hand, China is actually setting a course to become a world leader in AI. China’s leaders have made AI a strategic priority and have driven the Chinese tech industry to define standards and norms for global AI practices. However, in terms of citations per article, although China temporarily ranked first in the number of articles, there is still a long way to improve the overall quality of articles. By contrast, papers from France show high academic quality.

In our analytical study, the top 10 cited articles were considered the basis and reliable reference resources for further research. Along with the overlay visualization of co-occurring keywords and the timeline view of co-cited references, it revealed new research hotspots and what should be closely monitored.

Esophageal cancer (EC) is highly malignant, and the 5-year survival rate of advanced-stage EC is less than 25%,^[22]^ causing diagnostic, therapeutic, and financial burdens in high-risk regions. Studies have demonstrated AI’s diagnostic ability of AI to detect EC, including squamous cell carcinoma and adenocarcinoma.^[23]^ One of the latest AI tasks is to use DL techniques to detect both the early stages of esophageal squamous cell carcinoma and esophageal adenocarcinoma in Barrett’s esophagus. This finding was consistent with that of the largest research hotspot. Some studies have determined that compared to endoscopists, AI systems for Barrett’s esophagus targeted evaluation utilizing white-light endoscopy, volumetric laser endoscopy, and endomicroscopy^[24]^ have exhibited high-performance metrics. AI can also be used to monitor lymph node metastasis of EC and predict prognosis based on various demographic, clinicopathologic, hematologic, radiologic, and genetic variables,^[25]^ create a personalized real-time treatment regimen,^[26]^ and assess treatment response.^[27]^

AI in endoscopy has frequently been implemented in the stomach. Some researchers are currently using AI to determine Helicobacter pylori (HP) infections.^[28]^ A CNN diagnostic system trained on 32,208 HP-positive or-negative images illustrates the potential of AI in the endoscopic diagnosis of HP infection.^[29]^ The publication of gastric cancer ranked first with 142 citations, highlighting researchers’ concerns regarding AI in endoscopy for gastric cancer. The early diagnosis of digestive system tumors has always been a hot topic in the medical field. Many patients with gastric cancer are already at an advanced stage when detected, which predicts worse clinical prognosis. Digestive endoscopy is an effective means for the early detection of tumors, improving the 5-year survival rate of patients with malignant tumors and reducing mortality.^[30]^ However, early precancerous lesions of the digestive system are generally less involved, their depth is shallow, and the endoscopic morphology is not obvious. Thus, it is difficult to distinguish gastric mucosal lesions of early gastric cancer from the background mucosa. AI systems for diagnosing gastric cancer have achieved high accuracy, even if the lesion is less than 5 mm.^[31]^ Similar to esophageal lesions, assessing the depth of invasion of gastric cancer is an important management strategy. AI systems can distinguish between lesions that invade 500 μm or more below the mucosa and those that are more superficial.^[32]^ However, at present, there are no reports of large-scale application of AI for dynamic video detection of early cancer in the upper gastrointestinal tract, and existing studies have mostly focused on the image recognition stage. The main problems are poor specificity, high false-positive rate, and the small amount of data tested, which still need to be validated with a larger dataset.

Capsule endoscopy (CE)^[33,34]^ has been increasingly used for the evaluation of small bowel lesions, and “capsule endoscopy” was one of the most influential keywords. AI application in CE will help determine issues such as poor localization of the capsule, which may make CE more helpful for both small-bowel and colon evaluation. The most impressive example is the detection of protruding lesions in the small bowel.^[35]^ The identified lesions included polyps, nodules, epithelial tumors, and submucosal tumors. Other detection methods for individual lesions include angioectasias,^[36]^ erosions or ulcerations,^[37]^ and hookworms.^[38]^ This was a prospective study, although further validation is needed to determine whether CE can detect and characterize multiple lesions simultaneously.^[39]^

As the timeline visualization showed, #1 anatomical classification, #3 AI-based classification were related to polyp characterization, demonstrating that this is a current research hotspot and direction. Besides, “classification,” “ulcerative colitis,” and “polyp detection,” were noted as new research hotspots and should be closely monitored. Colorectal cancer is a common malignant tumor worldwide. According to global cancer statistics in 2021, it ranks third in incidence and second in mortality.^[40]^ Research data shows that colorectal transformation from normal mucosa to advanced malignant tumors will undergo multiple pathological processes, such as polyps, adenomas, intraepithelial neoplasia, and early cancer, with a period of 15 to 20 years. If screening can be performed during this period, the early detection of cancer can significantly reduce the morbidity and mortality of colorectal cancer. Nevertheless, polyps can be missed, with missing rates of up to 27%.^[41]^ There are many reasons for the missed diagnosis of polyps, such as poor intestinal preparation, inadequate mucosal examination, and lack of identification of subtle mucosal changes representing flat polyps. Consequently, the automatic detection of colorectal polyps has become one of the main areas of concern in the application of AI in gastrointestinal endoscopy. AI has recently been introduced to detect and classify polyps and adenomas, and this technique has shown encouraging outcomes in preliminary studies,^[42,43]^ which can effectively improve adenoma detection rate. One important finding was the excellent performance of AI compared with that of non-expert endoscopists on histology prediction. Endoscopic histology prediction requires substantial experience, but experienced endoscopists are not readily available. Hence, the availability of an AI-assisted system can potentially fill this gap. Currently, there are many studies on computer-aided diagnosis (CAD),^[44,45]^ and the detection methods are becoming more diversified. White-light endoscopy is the most basic and widely available diagnostic modality for endoscopy. Therefore, the application of CAD in white-light endoscopy is beneficial. A DL-based CAD study of colorectal lesions has been reported to distinguish adenomatous from non-adenomatous lesions.^[46]^ Nevertheless, owing to its relatively low accuracy, an additional algorithm is required. Narrow-band imaging or blue laser imaging allows detailed evaluation of the surface vessel and structural patterns. Studies of DL-based CAD for narrow-band imaging have been reported to differentiate between adenomas and hyperplastic polyps.^[47]^ Real-time working CAD can also be used to classify visualized polyps.^[48]^ Linked color imaging systems can produce clear and bright images using short-wavelength narrowband laser light. It is possible to distinguish between adenomatous and non-adenomatous polyps using a CAD system based on linked color images.^[49]^ Moreover, the application of AI in ultra-magnifying endoscopy has good prospects.^[50]^

## 5. Limitations

To the best of our knowledge, this bibliometric analytical study is the first to examine AI applications in digestive endoscopy. However, this study has some limitations. First, we considered the WoSCC database as a reputable and reliable service for publications and citations; hence, we extracted data only from it, limiting the coverage of all possible articles, which resulted in a smaller number of documents included in the analysis. Second, the search strategy might also have been insufficient, which may have led to a lack of articles due to other terminology. In addition, we selectively analyzed the information. Therefore, some crucial points and details may have been overlooked. In addition, non-AI experts may have difficulty in assessing publications on AI. Consequently, the authoritativeness of the studies included in this analytical study remains to be verified. All of the above-mentioned reasons may have led to a bias in the results. Hence, the results should be interpreted with caution.

## 6. Conclusion

AI is a promising technology for digestive endoscopy, particularly for lesion detection and polyp characterization. Further prospective in vivo studies are warranted in the future.

## Author contributions

**Conceptualization:** Xian Zhou, Xiaowei Tang.

**Methodology:** Xiaowei Tang.

**Supervision:** Mu-Han Lü, Xiaowei Tang.

**Visualization:** Pei-Ling Gan, Hui-Fang Xia.

**Writing – original draft:** Pei-Ling Gan.

**Writing – review & editing:** Shu Huang, Xiao Pan.

## Supplementary Material
