# Supplementary material for: The scientific progress and prospects of artificial intelligence in digestive endoscopy: A comprehensive bibliometric analysis
Source: Medicine (Baltimore). 2022 Nov 25;101(47):e31931. doi: 10.1097/MD.0000000000031931 (PMC9704924; doi:10.1097/MD.0000000000031931)
Supplement: Supplementary file 1 [file medi-101-e31931-s001.pdf]

Table S1 The top-100 cited articles in the field of artificial intelligence in digestive endoscopy field.

Rank	Article	Number of citations
1	Hirasawa T, Aoyama K, Tanimoto T, et al. Application of artificial intelligence using a convolutional neural network for detecting gastric cancer in endoscopic images. *Gastric Cancer*. 2018;21(4):653-660. doi:10.1007/s10120-018-0793-2	255
2	Wang P, Berzin TM, Glissen Brown JR, et al. Real-time automatic detection system increases colonoscopic polyp and adenoma detection rates: a prospective randomised controlled study. *Gut*. 2019;68(10):1813-1819. doi:10.1136/gutjnl-2018-317500	248
3	Mori Y, Kudo SE, Misawa M, et al. Real-Time Use of Artificial Intelligence in Identification of Diminutive Polyps During Colonoscopy: A Prospective Study. *Ann Intern Med*. 2018;169(6):357-366. doi:10.7326/M18-0249	195
4	Horie Y, Yoshio T, Aoyama K, et al. Diagnostic outcomes of esophageal cancer by artificial intelligence using convolutional neural networks. *Gastrointest Endosc*. 2019;89(1):25-32. doi:10.1016/j.gie.2018.07.037	153
5	Le Berre C, Sandborn WJ, Aridhi S, et al. Application of Artificial Intelligence to Gastroenterology and Hepatology. *Gastroenterology*. 2020;158(1):76-94.e2. doi:10.1053/j.gastro.2019.08.058	129
6	Zhu Y, Wang QC, Xu MD, et al. Application of convolutional neural network in the diagnosis of the invasion depth of gastric cancer based on conventional endoscopy. *Gastrointest Endosc*. 2019;89(4):806-815.e1.	127

	doi:10.1016/j.gie.2018.11.011	
7	Shichijo S, Nomura S, Aoyama K, et al. Application of Convolutional Neural Networks in the Diagnosis of Helicobacter pylori Infection Based on Endoscopic Images. *EBioMedicine*. 2017;25:106-111. doi:10.1016/j.ebiom.2017.10.014	124
8	Luo H, Xu G, Li C, et al. Real-time artificial intelligence for detection of upper gastrointestinal cancer by endoscopy: a multicentre, case-control, diagnostic study. *Lancet Oncol*. 2019;20(12):1645-1654. doi:10.1016/S1470-2045(19)30637-0	110
9	Aoki T, Yamada A, Aoyama K, et al. Automatic detection of erosions and ulcerations in wireless capsule endoscopy images based on a deep convolutional neural network. *Gastrointest Endosc*. 2019;89(2):357-363.e2. doi:10.1016/j.gie.2018.10.027	110
10	Wu L, Zhang J, Zhou W, et al. Randomised controlled trial of WISENSE, a real-time quality improving system for monitoring blind spots during esophagogastroduodenoscopy. *Gut*. 2019;68(12):2161-2169. doi:10.1136/gutjnl-2018-317366	97
11	Repici A, Badalamenti M, Maselli R, et al. Efficacy of Real-Time Computer-Aided Detection of Colorectal Neoplasia in a Randomized Trial. *Gastroenterology*. 2020;159(2):512-520.e7. doi:10.1053/j.gastro.2020.04.062	96
12	Ding Z, Shi H, Zhang H, et al. Gastroenterologist-Level Identification of Small-Bowel Diseases and Normal Variants by Capsule Endoscopy Using a Deep-Learning Model. *Gastroenterology*. 2019;157(4):1044-1054.e5. doi:10.1053/j.gastro.2019.06.025	95

13	de Groof AJ, Struyvenberg MR, van der Putten J, et al. Deep-Learning System Detects Neoplasia in Patients With Barrett's Esophagus With Higher Accuracy Than Endoscopists in a Multistep Training and Validation Study With Benchmarking. *Gastroenterology*. 2020;158(4):915-929.e4. doi:10.1053/j.gastro.2019.11.030	90
14	Yuan Y, Meng MQ. Deep learning for polyp recognition in wireless capsule endoscopy images. *Med Phys*. 2017;44(4):1379-1389. doi:10.1002/mp.12147	86
15	Itoh T, Kawahira H, Nakashima H, Yata N. Deep learning analyzes Helicobacter pylori infection by upper gastrointestinal endoscopy images. *Endosc Int Open*. 2018;6(2):E139-E144. doi:10.1055/s-0043-120830	77
16	Bisschops R, East JE, Hassan C, et al. Advanced imaging for detection and differentiation of colorectal neoplasia: European Society of Gastrointestinal Endoscopy (ESGE) Guideline - Update 2019 [published correction appears in Endoscopy. 2019 Dec;51(12):C6]. *Endoscopy*. 2019;51(12):1155-1179. doi:10.1055/a-1031-7657	76
17	Maeda Y, Kudo SE, Mori Y, et al. Fully automated diagnostic system with artificial intelligence using endocytoscopy to identify the presence of histologic inflammation associated with ulcerative colitis (with video). *Gastrointest Endosc*. 2019;89(2):408-415. doi:10.1016/j.gie.2018.09.024	72
18	Iakovidis DK, Georgakopoulos SV, Vasilakakis M, Koulaouzidis A, Plagianakos VP. Detecting and Locating Gastrointestinal Anomalies Using Deep Learning and Iterative Cluster Unification. *IEEE Trans Med Imaging*. 2018;37(10):2196-2210.	71

	doi:10.1109/TMI.2018.2837002	
19	Alagappan M, Brown JRG, Mori Y, Berzin TM. Artificial intelligence in gastrointestinal endoscopy: The future is almost here. *World J Gastrointest Endosc*. 2018;10(10):239-249. doi:10.4253/wjge.v10.i10.239	65
20	Yang YJ, Bang CS. Application of artificial intelligence in gastroenterology. *World J Gastroenterol*. 2019;25(14):1666-1683. doi:10.3748/wjg.v25.i14.1666	64
21	Kudo SE, Misawa M, Mori Y, et al. Artificial Intelligence-assisted System Improves Endoscopic Identification of Colorectal Neoplasms. *Clin Gastroenterol Hepatol*. 2020;18(8):1874-1881.e2. doi:10.1016/j.cgh.2019.09.009	64
22	Nakashima H, Kawahira H, Kawachi H, Sakaki N. Artificial intelligence diagnosis of *Helicobacter pylori* infection using blue laser imaging-bright and linked color imaging: a single-center prospective study. *Ann Gastroenterol*. 2018;31(4):462-468. doi:10.20524/aog.2018.0269	62
23	Li L, Chen Y, Shen Z, et al. Convolutional neural network for the diagnosis of early gastric cancer based on magnifying narrow band imaging. *Gastric Cancer*. 2020;23(1):126-132. doi:10.1007/s10120-019-00992-2	60
24	Khan, M. , et al. "Gastrointestinal Diseases Segmentation and Classification based on Duo-Deep Architectures." Pattern Recognition Letters 131(2019).	59
25	Vinsard DG, Mori Y, Misawa M, et al. Quality assurance of computer-aided detection and diagnosis in colonoscopy. *Gastrointest Endosc*. 2019;90(1):55-63. doi:10.1016/j.gie.2019.03.019	57

26	Soffer S, Klang E, Shimon O, et al. Deep learning for wireless capsule endoscopy: a systematic review and meta-analysis. *Gastrointest Endosc*. 2020;92(4):831-839.e8. doi:10.1016/j.gie.2020.04.039	55
27	Kudo SE, Mori Y, Misawa M, et al. Artificial intelligence and colonoscopy: Current status and future perspectives. *Dig Endosc*. 2019;31(4):363-371. doi:10.1111/den.13340	53
28	Tsuboi A, Oka S, Aoyama K, et al. Artificial intelligence using a convolutional neural network for automatic detection of small-bowel angioectasia in capsule endoscopy images. *Dig Endosc*. 2020;32(3):382-390. doi:10.1111/den.13507	53
29	Khan, M. A. , et al. "Computer-Aided Gastrointestinal Diseases Analysis from Wireless Capsule Endoscopy: A Framework of Best Features Selection." IEEE Access PP.99(2020).	51
30	Cai SL, Li B, Tan WM, et al. Using a deep learning system in endoscopy for screening of early esophageal squamous cell carcinoma (with video). *Gastrointest Endosc*. 2019;90(5):745-753.e2. doi:10.1016/j.gie.2019.06.044	51
31	Khan MA, Sharif M, Akram T, Yasmin M, Nayak RS. Stomach Deformities Recognition Using Rank-Based Deep Features Selection. *J Med Syst*. 2019;43(12):329. Published 2019 Nov 1. doi:10.1007/s10916-019-1466-3	50
32	Min JK, Kwak MS, Cha JM. Overview of Deep Learning in Gastrointestinal Endoscopy. *Gut Liver*. 2019;13(4):388-393. doi:10.5009/gnl18384	50
33	Ruffle JK, Farmer AD, Aziz Q. Artificial Intelligence-Assisted Gastroenterology-Promises and Pitfalls. *Am J Gastroenterol*. 2019;114(3):422-428.	50

	doi:10.1038/s41395-018-0268-4	
34	Ohmori M, Ishihara R, Aoyama K, et al. Endoscopic detection and differentiation of esophageal lesions using a deep neural network. *Gastrointest Endosc*. 2020;91(2):301-309.e1. doi:10.1016/j.gie.2019.09.034	46
35	de Groof J, van der Sommen F, van der Putten J, et al. The Argos project: The development of a computer-aided detection system to improve detection of Barrett's neoplasia on white light endoscopy. *United European Gastroenterol J*. 2019;7(4):538-547. doi:10.1177/2050640619837443	46
36	Horiuchi Y, Aoyama K, Tokai Y, et al. Convolutional Neural Network for Differentiating Gastric Cancer from Gastritis Using Magnified Endoscopy with Narrow Band Imaging. *Dig Dis Sci*. 2020;65(5):1355-1363. doi:10.1007/s10620-019-05862-6	41
37	Yoon HJ, Kim S, Kim JH, et al. A Lesion-Based Convolutional Neural Network Improves Endoscopic Detection and Depth Prediction of Early Gastric Cancer. *J Clin Med*. 2019;8(9):1310. Published 2019 Aug 26. doi:10.3390/jcm8091310	38
38	Billah M, Waheed S, Rahman MM. An Automatic Gastrointestinal Polyp Detection System in Video Endoscopy Using Fusion of Color Wavelet and Convolutional Neural Network Features. *Int J Biomed Imaging*. 2017;2017:9545920. doi:10.1155/2017/9545920	36
39	Aoki T, Yamada A, Kato Y, et al. Automatic detection of blood content in capsule endoscopy images based on a deep convolutional neural network. *J Gastroenterol Hepatol*. 2020;35(7):1196-1200. doi:10.1111/jgh.14941	36

40	Wang P, Liu P, Glissen Brown JR, et al. Lower Adenoma Miss Rate of Computer-Aided Detection-Assisted Colonoscopy vs Routine White-Light Colonoscopy in a Prospective Tandem Study. *Gastroenterology*. 2020;159(4):1252-1261.e5. doi:10.1053/j.gastro.2020.06.023	35
41	Everson M, Herrera L, Li W, et al. Artificial intelligence for the real-time classification of intrapapillary capillary loop patterns in the endoscopic diagnosis of early oesophageal squamous cell carcinoma: A proof-of-concept study. *United European Gastroenterol J*. 2019;7(2):297-306. doi:10.1177/2050640618821800	35
42	Borgli H, Thambawita V, Smedsrud PH, et al. HyperKvasir, a comprehensive multi-class image and video dataset for gastrointestinal endoscopy. *Sci Data*. 2020;7(1):283. Published 2020 Aug 28. doi:10.1038/s41597-020-00622-y	34
43	Khan MA, Sharif M, Akram T, Yasmin M, Nayak RS. Stomach Deformities Recognition Using Rank-Based Deep Features Selection. *J Med Syst*. 2019;43(12):329. Published 2019 Nov 1. doi:10.1007/s10916-019-1466-3	34
44	Lui TKL, Guo CG, Leung WK. Accuracy of artificial intelligence on histology prediction and detection of colorectal polyps: a systematic review and meta-analysis. *Gastrointest Endosc*. 2020;92(1):11-22.e6. doi:10.1016/j.gie.2020.02.033	34
45	Ozawa T, Ishihara S, Fujishiro M, Kumagai Y, Shichijo S, Tada T. Automated endoscopic detection and classification of colorectal polyps using convolutional neural networks. *Therap Adv Gastroenterol*. 2020;13:1756284820910659. Published 2020 Mar 20. doi:10.1177/1756284820910659	33
46	Zhou J, Wu L, Wan X, et al. A novel artificial intelligence system for the assessment	33

	of bowel preparation (with video). *Gastrointest Endosc*. 2020;91(2):428-435.e2. doi:10.1016/j.gie.2019.11.026	
47	Lee JH, Kim YJ, Kim YW, et al. Spotting malignancies from gastric endoscopic images using deep learning. *Surg Endosc*. 2019;33(11):3790-3797. doi:10.1007/s00464-019-06677-2	32
48	Aoki T, Yamada A, Aoyama K, et al. Clinical usefulness of a deep learning-based system as the first screening on small-bowel capsule endoscopy reading. *Dig Endosc*. 2020;32(4):585-591. doi:10.1111/den.13517	32
49	Kaul V, Enslin S, Gross SA. History of artificial intelligence in medicine. *Gastrointest Endosc*. 2020;92(4):807-812. doi:10.1016/j.gie.2020.06.040	31
50	van der Sommen F, de Groof J, Struyvenberg M, et al. Machine learning in GI endoscopy: practical guidance in how to interpret a novel field. *Gut*. 2020;69(11):2035-2045. doi:10.1136/gutjnl-2019-320466	31
51	Shichijo S, Endo Y, Aoyama K, et al. Application of convolutional neural networks for evaluating Helicobacter pylori infection status on the basis of endoscopic images. *Scand J Gastroenterol*. 2019;54(2):158-163. doi:10.1080/00365521.2019.1577486	29
52	Zhang Y, Li F, Yuan F, et al. Diagnosing chronic atrophic gastritis by gastroscopy using artificial intelligence. *Dig Liver Dis*. 2020;52(5):566-572. doi:10.1016/j.dld.2019.12.146	28
53	Chen D, Wu L, Li Y, et al. Comparing blind spots of unsedated ultrafine, sedated, and unsedated conventional gastroscopy with and without artificial intelligence: a	28

	prospective, single-blind, 3-parallel-group, randomized, single-center trial. *Gastrointest Endosc*. 2020;91(2):332-339.e3. doi:10.1016/j.gie.2019.09.016	
54	Takagi, R., R. Horisaki, and J. Tanida . "Object recognition through a multi-mode fiber." Optical Review 24.2(2017):117-120.	27
55	Lui TKL, Tsui VWM, Leung WK. Accuracy of artificial intelligence-assisted detection of upper GI lesions: a systematic review and meta-analysis. *Gastrointest Endosc*. 2020;92(4):821-830.e9. doi:10.1016/j.gie.2020.06.034	26
56	Ciuti G, Skonieczna-Żydecka K, Marlicz W, et al. Frontiers of Robotic Colonoscopy: A Comprehensive Review of Robotic Colonoscopes and Technologies. *J Clin Med*. 2020;9(6):1648. Published 2020 May 31. doi:10.3390/jcm9061648	25
57	Ded, A, A. Dki , and B. Ak . "Look-behind fully convolutional neural network for computer-aided endoscopy." Biomedical Signal Processing and Control 49(2019):192-201.	25
58	Ang TL, Carneiro G. Artificial intelligence in gastrointestinal endoscopy. *J Gastroenterol Hepatol*. 2021;36(1):5-6. doi:10.1111/jgh.15344	25
59	Ikenoyama Y, Hirasawa T, Ishioka M, et al. Detecting early gastric cancer: Comparison between the diagnostic ability of convolutional neural networks and endoscopists. *Dig Endosc*. 2021;33(1):141-150. doi:10.1111/den.13688	25
60	Hosoe N, Takabayashi K, Ogata H, Kanai T. Capsule endoscopy for small-intestinal disorders: Current status. *Dig Endosc*. 2019;31(5):498-507. doi:10.1111/den.13346	24
61	Mahmood F, Chen R, Sudarsky S, Yu D, Durr NJ. Deep learning with cinematic rendering: fine-tuning deep neural networks using photorealistic medical	24

	images. *Phys Med Biol*. 2018;63(18):185012. Published 2018 Sep 13. doi:10.1088/1361-6560/aada93	
62	Zheng W, Zhang X, Kim JJ, et al. High Accuracy of Convolutional Neural Network for Evaluation of Helicobacter pylori Infection Based on Endoscopic Images: Preliminary Experience. *Clin Transl Gastroenterol*. 2019;10(12):e00109. doi:10.14309/ctg.0000000000000109	24
63	Ueyama H, Kato Y, Akazawa Y, et al. Application of artificial intelligence using a convolutional neural network for diagnosis of early gastric cancer based on magnifying endoscopy with narrow-band imaging. *J Gastroenterol Hepatol*. 2021;36(2):482-489. doi:10.1111/jgh.15190	23
64	de Souza LA Jr, Palm C, Mendel R, et al. A survey on Barrett's esophagus analysis using machine learning. *Comput Biol Med*. 2018;96:203-213. doi:10.1016/j.compbiomed.2018.03.014	21
65	Owais M, Arsalan M, Choi J, Mahmood T, Park KR. Artificial Intelligence-Based Classification of Multiple Gastrointestinal Diseases Using Endoscopy Videos for Clinical Diagnosis. *J Clin Med*. 2019;8(7):986. Published 2019 Jul 7. doi:10.3390/jcm8070986	21
66	Wang, Hao, et al. "Smart connected electronic gastroscope system for gastric cancer screening using multi-column convolutional neural networks." International Journal of Production Research (2018):1-12.	21
67	Gulati S, Patel M, Emmanuel A, Haji A, Hayee B, Neumann H. The future of endoscopy: Advances in endoscopic image innovations. *Dig Endosc*. 2020;32(4):512-	20

	522. doi:10.1111/den.13481	
68	Ebigbo A, Palm C, Probst A, et al. A technical review of artificial intelligence as applied to gastrointestinal endoscopy: clarifying the terminology. *Endosc Int Open*. 2019;7(12):E1616-E1623. doi:10.1055/a-1010-5705	20
69	Cogan T, Cogan M, Tamil L. MAPGI: Accurate identification of anatomical landmarks and diseased tissue in gastrointestinal tract using deep learning. *Comput Biol Med*. 2019;111:103351. doi:10.1016/j.compbiomed.2019.103351	20
70	El Hajjar A, Rey JF. Artificial intelligence in gastrointestinal endoscopy: general overview. *Chin Med J (Engl)*. 2020;133(3):326-334. doi:10.1097/CM9.0000000000000623	19
71	Eminaga O, Eminaga N, Semjonow A, Breil B. Diagnostic Classification of Cystoscopic Images Using Deep Convolutional Neural Networks. *JCO Clin Cancer Inform*. 2018;2:1-8. doi:10.1200/CCI.17.00126	19
72	Lan, L., et al. "Deep Convolutional Neural Networks for WCE Abnormality Detection: CNN Architecture, Region Proposal and Transfer Learning." IEEE Access (2019):1-1. doi:10.1109/ACCESS.2019.2901568	18
73	van der Sommen F, Curvers WL, Nagengast WB. Novel Developments in Endoscopic Mucosal Imaging. *Gastroenterology*. 2018;154(7):1876-1886. doi:10.1053/j.gastro.2018.01.070	18
74	Chen, Honghan, et al. "Automatic content understanding with cascaded spatial-temporal deep framework for capsule endoscopy videos." Neurocomputing 229.MAR.15(2017):77-87. Doi : 10.1016/j.neucom.2016.06.077	18

75	Yao H, Najarian K, Gryak J, et al. Fully automated endoscopic disease activity assessment in ulcerative colitis. *Gastrointest Endosc*. 2021;93(3):728-736.e1. doi:10.1016/j.gie.2020.08.011	18
76	Pace F, Buscema M, Dominici P, et al. Artificial neural networks are able to recognize gastro-oesophageal reflux disease patients solely on the basis of clinical data. *Eur J Gastroenterol Hepatol*. 2005;17(6):605-610. doi:10.1097/00042737-200506000-00003	18
77	Luo X, Mori K, Peters TM. Advanced Endoscopic Navigation: Surgical Big Data, Methodology, and Applications. *Annu Rev Biomed Eng*. 2018;20:221-251. doi:10.1146/annurev-bioeng-062117-120917	17
78	Du, W. , et al. "Review on the Applications of Deep Learning in the Analysis of Gastrointestinal Endoscopy Images." IEEE Access PP.99(2019):1-1.doi: 10.1109/ACCESS.2019.2944676	17
79	Niu PH, Zhao LL, Wu HL, Zhao DB, Chen YT. Artificial intelligence in gastric cancer: Application and future perspectives. *World J Gastroenterol*. 2020;26(36):5408-5419. doi:10.3748/wjg.v26.i36.5408	17
80	Jin P, Ji X, Kang W, et al. Artificial intelligence in gastric cancer: a systematic review. *J Cancer Res Clin Oncol*. 2020;146(9):2339-2350. doi:10.1007/s00432-020-03304-9	17
81	Dray X, Iakovidis D, Houdeville C, et al. Artificial intelligence in small bowel capsule endoscopy - current status, challenges and future promise. *J Gastroenterol Hepatol*. 2021;36(1):12-19. doi:10.1111/jgh.15341	17

82	Elena RM, Riccardo U, Rossella C, Bizzotto A, Domenico G, Guido C. Current status of device-assisted enteroscopy: Technical matters, indication, limits and complications. *World J Gastrointest Endosc*. 2012;4(10):453-461. doi:10.4253/wjge.v4.i10.453	16
83	Choi J, Shin K, Jung J, et al. Convolutional Neural Network Technology in Endoscopic Imaging: Artificial Intelligence for Endoscopy. *Clin Endosc*. 2020;53(2):117-126. doi:10.5946/ce.2020.054	16
84	Wang S, Xing Y, Zhang L, Gao H, Zhang H. Deep Convolutional Neural Network for Ulcer Recognition in Wireless Capsule Endoscopy: Experimental Feasibility and Optimization. *Comput Math Methods Med*. 2019;2019:7546215. Published 2019 Sep 18. doi:10.1155/2019/7546215	16
85	Bang CS, Lee JJ, Baik GH. Artificial Intelligence for the Prediction of Helicobacter Pylori Infection in Endoscopic Images: Systematic Review and Meta-Analysis Of Diagnostic Test Accuracy. *J Med Internet Res*. 2020;22(9):e21983. Published 2020 Sep 16. doi:10.2196/21983	16
86	Nakashima H, Kawahira H, Kawachi H, Sakaki N. Endoscopic three-categorical diagnosis of Helicobacter pylori infection using linked color imaging and deep learning: a single-center prospective study (with video). *Gastric Cancer*. 2020;23(6):1033-1040. doi:10.1007/s10120-020-01077-1	16
87	Mori Y, Neumann H, Misawa M, Kudo SE, Bretthauer M. Artificial intelligence in colonoscopy - Now on the market. What's next?. *J Gastroenterol Hepatol*. 2021;36(1):7-11. doi:10.1111/jgh.15339	16

88	Hwang Y, Park J, Lim YJ, Chun HJ. Application of Artificial Intelligence in Capsule Endoscopy: Where Are We Now?. *Clin Endosc*. 2018;51(6):547-551. doi:10.5946/ce.2018.173	15
89	Igarashi S, Sasaki Y, Mikami T, Sakuraba H, Fukuda S. Anatomical classification of upper gastrointestinal organs under various image capture conditions using AlexNet. *Comput Biol Med*. 2020;124:103950. doi:10.1016/j.compbiomed.2020.103950	15
90	Otani K, Nakada A, Kurose Y, et al. Automatic detection of different types of small-bowel lesions on capsule endoscopy images using a newly developed deep convolutional neural network. *Endoscopy*. 2020;52(9):786-791. doi:10.1055/a-1167-8157	15
91	Marlicz W, Ren X, Robertson A, et al. Frontiers of Robotic Gastroscopy: A Comprehensive Review of Robotic Gastroscopes and Technologies. *Cancers (Basel)*. 2020;12(10):2775. Published 2020 Sep 28. doi:10.3390/cancers12102775	14
92	Iakovidis DK, Dimas G, Karargyris A, Bianchi F, Ciuti G, Koulaouzidis A. Deep Endoscopic Visual Measurements. *IEEE J Biomed Health Inform*. 2019;23(6):2211-2219. doi:10.1109/JBHI.2018.2853987	14
93	He YS, Su JR, Li Z, Zuo XL, Li YQ. Application of artificial intelligence in gastrointestinal endoscopy. *J Dig Dis*. 2019;20(12):623-630. doi:10.1111/1751-2980.12827	14
94	Wang S, Xing Y, Zhang L, Gao H, Zhang H. A systematic evaluation and optimization of automatic detection of ulcers in wireless capsule endoscopy on a large	14

	dataset using deep convolutional neural networks. *Phys Med Biol*. 2019;64(23):235014. Published 2019 Dec 5. doi:10.1088/1361-6560/ab5086	
95	Parasa S, Wallace M, Bagci U, et al. Proceedings from the First Global Artificial Intelligence in Gastroenterology and Endoscopy Summit. *Gastrointest Endosc*. 2020;92(4):938-945.e1. doi:10.1016/j.gie.2020.04.044	14
96	Okagawa Y, Abe S, Yamada M, Oda I, Saito Y. Artificial Intelligence in Endoscopy [published online ahead of print, 2021 Jun 21]. *Dig Dis Sci*. 2021;10.1007/s10620-021-07086-z. doi:10.1007/s10620-021-07086-z	14
97	Ward TM, Hashimoto DA, Ban Y, et al. Automated operative phase identification in peroral endoscopic myotomy. *Surg Endosc*. 2021;35(7):4008-4015. doi:10.1007/s00464-020-07833-9	14
98	Muhammad, K., et al. "Vision-based personalized Wireless Capsule Endoscopy for smart healthcare: Taxonomy, literature review, opportunities and challenges." Future Generation Computer Systems 113(2020). Doi:10.1016/j.future.2020.06.048	13
99	Dohi O, Majima A, Naito Y, et al. Can image-enhanced endoscopy improve the diagnosis of Kyoto classification of gastritis in the clinical setting?. *Dig Endosc*. 2020;32(2):191-203. doi:10.1111/den.13540	13
100	Arribas J, Antonelli G, Frazzoni L, et al. Standalone performance of artificial intelligence for upper GI neoplasia: a meta-analysis [published online ahead of print, 2020 Oct 30]. *Gut*. 2020;gutjnl-2020-321922. doi:10.1136/gutjnl-2020-321922	13
