# Supplementary material for: The scientific progress and prospects of artificial intelligence in digestive endoscopy: A comprehensive bibliometric analysis
Source: Medicine (Baltimore). 2022 Nov 25;101(47):e31931. doi: 10.1097/MD.0000000000031931 (PMC9704924; doi:10.1097/MD.0000000000031931)
Supplement: Supplementary file 2 [file medi-101-e31931-s002.pdf]

Table S2 All countries of retrieved articles in the field of artificial intelligence in digestive endoscopy.

Rank	Country	Number of articles	Number of citations
1	China	136	1638
2	USA	110	1316
3	Japan	75	2040
4	England	58	593
5	Germany	49	487
6	Italy	47	530
7	South Korea	43	437
8	Portugal	25	77
9	France	22	406
10	Netherlands	22	327
11	India	19	231
12	Norway	18	165
13	Canada	17	90
14	Spain	16	141
15	Pakistan	15	233
16	Scotland	14	195
17	Belgium	13	243
18	Australia	12	141
19	Poland	12	133

20	Sweden	11	181
21	Greece	11	146
22	Israel	10	85
23	Austria	8	48
24	Singapore	8	33
25	Saudi arabia	7	110
26	Egypt	7	31
27	Denmark	7	29
28	Romania	7	16
29	Ireland	7	12
30	Bangladesh	6	63
31	Switzerland	6	26
32	Lebanon	5	135
33	Brazil	5	47
34	Russia	5	37
35	Vietnam	4	57
36	Turkey	4	22
37	Malaysia	4	7
38	United Arab Emirates	3	3
39	Kuwait	2	96
40	Iran	2	10
41	Malta	2	6

42	Colombia	2	2
43	Morocco	1	8
44	Finland	1	4
45	Hungary	1	1
46	Qatar	1	1
47	Czech Republic	1	0
48	Ecuador	1	0
49	Iraq	1	0
50	Philippines	1	0
51	Wales	1	0
